# Detection and Molecular Characterization of J Subgroup Avian Leukosis Virus in Wild Ducks in China

**DOI:** 10.1371/journal.pone.0094980

**Published:** 2014-04-14

**Authors:** Xiangwei Zeng, Lanlan Liu, Ruijun Hao, Chunyan Han

**Affiliations:** 1 College of Wildlife Resources, Northeast Forestry University, Harbin, China; 2 College of Basic Medical Science, Heilongjiang University of Chinese Medicine, Harbin, China; University of Maryland, United States of America

## Abstract

To assess the status of avian leukosis virus subgroup J (ALV-J) in wild ducks in China, we examined samples from 528 wild ducks, representing 17 species, which were collected in China over the past 3 years. Virus isolation and PCR showed that 7 ALV-J strains were isolated from wild ducks. The env genes and the 3′UTRs from these isolates were cloned and sequenced. The env genes of all 7 wild duck isolates were significantly different from those in the prototype strain HPRS-103, American strains, broiler ALV-J isolates and Chinese local chicken isolates, but showed close homology with those found in some layer chicken ALV-J isolates and belonged to the same group. The 3′UTRs of 7 ALV-J wild ducks isolates showed close homology with the prototype strain HPRS-103 and no obvious deletion was found in the 3′UTR except for a 1 bp deletion in the E element that introduced a binding site for c-Ets-1. Our study demonstrated the presence of ALV-J in wild ducks and investigated the molecular characterization of ALV-J in wild ducks isolates.

## Introduction

Avian leukosis virus subgroup J (ALV-J) was first isolated from meat-type chickens in the late 1980s in the United Kingdom [1]. All of the ALVs are currently classified into 10 subgroups (A to J) according to host range, viral envelope interference and cross-neutralization patterns [2]. ALV-J has caused more serious damage than has been caused by any other subgroup, and it has been widely recognized and reported in many regions of the world [3]–[5].Over the past decade or so, the host range of ALV-J has clearly increased, infecting meat-type chickens and then layer flocks and many local chickens in China.

It is urgent and necessary for us to research the molecular epidemiology of ALV-J to further understand the continuous evolution of this virus. Past molecular epidemiology studies of ALV-J were mainly focused on poultry [6]–[8], and reports of infection in wild birds were rare. ALVs in earlier studies were not subgroup-specific. Neumann reported the work from the 1980s and 1990s on ALV in budgerigars and the possible causative link with renal tumours [9]. Dimcheff detected the ALV gag gene in 26 species of galliform birds [10]. As we known, four unfamiliar subgroups (F, G, H and I) of ALVs were isolated from wild fowl (e.g., pheasant and quail)[11]. Recently study demonstrated that ALV-A and ALV-B infections occured in the wild birds of Northeast China [12]. But in general, the scientific literatures on ALV in wild birds were limited.

Our study represents the report of ALV-J strains in wild ducks, and we sequenced and analyzed the env genes and 3′ untranslated regions (3′UTR) of ALV-J isolates from wild ducks to begin to elucidate the epidemiology of avian leukosis in wild birds in China.

## Materials and Methods

### Ethics Statement

All animal studies were approved by the Animal Welfare Committee of College of Wildlife Resources, Northeast Forestry University. All animal procedures were carried out in strict accordance with the international standards for animal welfare.

### Clinical samples

The collection of wild ducks samples is organized and managed by National Terrestrial Wildlife Pathogen-origins and Epidemic Diseases Monitoring Master Stations of State Forestry Administration. There are dozens of monitoring substations locating in every province of China, which are responsible for collecting samples of wild ducks and sending to some universities and research institutions for testing and research. At present, these substations collected samples through looking for dead wild ducks from nature reserve areas and bird banding stations, rather than capturing wild ducks alive. So the sample collection work did not cause harm to wildlife. In this study, a total of 528 dead wild ducks collected from National Terrestrial Wildlife Pathogen-origins and Epidemic Diseases Monitoring Stations in 5 provinces in China (Neimeng, Jilin, Heilongjiang, and Liaoning, Jiangxi) between April 2010 and September 2013 were sent to our Laboratory for diagnosis. In most cases, the cause of death was presumed to be either excessive struggling in nets during banding, pesticide poisoning, or some other unknown reasons. Samples of the spleen, kidney, liver, and other tissues from the dead ducks were collected and maintained at −80°C in our laboratory. The data from the wild ducks samples that were tested in the current study is shown in Table 1.

**Table 1:** Samples of wild ducks to test for ALV-J.

Order	Family	Species		Number of Samples	Number of Isolates
		English name	Latin name		
*Anseriformes*	*Anatidae*	Eurasian Wigeon	*Anas Penelope*	36	2
		Tufted Duck	*Aythya fuligula*	22	0
		Baer's Pochard	*Aythya baeri*	31	0
		Pintail	*Anas acuta*	15	0
		Mallard	*Anas platyrhynchos*	79	0
		Northern Shoveller	*Anas clypeata*	19	1
		Red-crested Pochard	*Rhodonessa rufina*	9	0
		Ruddy Shelduck	*Tadorna ferruginea*	25	0
		Common Goldeneye	*Bucephala clangula*	27	0
		Green-winged Teal	*Anas crecca*	75	2
		Baikal Teal	*Anas formosa*	67	2
		Falcated Duck	*Anas falcata*	29	0
		Mandarin Duck	*Aix galericulata*	8	0
		Garganey	*Anas querquedula*	17	0
		Gadwall	*Anas strepera*	21	0
		Spot-billed Duck	*Anas poecilorhyncha*	39	0
		Common Shelduck	*Tadorna tadorna*	9	0
1	1	17		528	7

### Virus isolation and proviral DNA extraction

Virus isolations were performed in DF-1 cells. The procedures for the isolation and identification of ALV in cell cultures were performed according to previously described studies [6], [12]. Briefly, filtered tissue homogenates were inoculated with DF-1 cells, which were cultured for two serial passages at 37°C in a 5% CO_2_ incubator with daily monitoring. The infected DF-1 cells were tested for the ALV group-specific antigen (p27) by an antigen capture enzyme-linked immunosorbent assay (AC-ELISA) on anti-p27 antibody-coated plates (IDEXX Inc., MA). The positive samples detected by ELISA were harvested for DNA extraction and PCR amplification. The DNA was directly extracted from the positive DF-1 cells using a Universal Genomic DNA Extraction Kit (TaKaRa, Dalian, China). Subsequently, the DNA was resuspended in nuclease-free water and stored at −80°C.

### PCR cloning and sequencing

PCR was used to test genomic DNA from the cultured DF-1 cells for the presence of envelope sequences that are specific for ALV-J, as previously described [13]. The primer set H5 (5′-GGATGAGGTGACTAAGA-3′) and H7 (5′-CGAACCAAAGGTAACACACG-3′) was used for the specific detection of ALV-J proviral DNA, which generates a 545 bp PCR product [13]. The primer set H5 and AD1 (5′-GGGAGGTGGCTGACTGTGT-3′) was used for the detection of ALV subgroups A to E, which generate a 295 to 326 bp PCR product [13]. Using the sequence of the ALV-J prototype strain HPRS-103 (Genbank accession number Z46390), the primer pair ALVJE+UF (5′-CGACACTGATAAGGTTATTTGGGT-3′) and ALVJE+UR (5′-TCGGAACCTACAGCTGCTC-3′) was designed for the amplification of a 2259 bp fragment that encompasses the entire ALV-J env gene and 3′UTR, including a nonfunctional redundant transmembrane region [rTM], the direct repeat element [DR-1], and an E element. The PCR conditions included an initial denaturation cycle of 4 min at 94°C followed by 30 cycles of denaturation for 1 min at 94°C, annealing for 1 min at 58°C, and an extension for 2.5 min at 72°C, with a final extension of 10 min at 72°C. All PCRs were carried out with PrimerSTAR HS DNA high-fidelity polymerase (TaKaRa, Dalian, China).

The PCR products were excised from a 1.0% agarose gel, purified using an AxyPrep DNA gel extraction kit (Axygen Scientific, Inc., CA), and cloned into the pMD18-T TA vector (TaKaRa). Three independent clones of each ALV-J isolate were sequenced by the Beijing Genomics Institute (Beijing, China).

### DNA alignments and phylogenetic analysis

The nucleotide sequences were aligned using Clustal W, version 1.8 [14]. A neighbor-joining tree was drawn using MEGA, version 4.0 [15], with confidence levels assessed using 1,000 bootstrap replications. The sequences obtained in this study have been submitted to Genbank. The Genbank sequences of the ALV-J strains that were isolated from broilers, layer chickens and Chinese local chickens were included in the multiple sequence alignment and phylogenetic analysis, and they are summarized in Table 2.

**Table 2:** ALV-J strains used in the construction of phylogenetic trees.

No.^a^	Strains	Country	Origin host	Accession No.
1	WB11008j	China	Baikal Teal	JX570785
2	WB11016j	China	Eurasian Wigeon	JX570795
3	WB11038j	China	Eurasian Wigeon	JX570789
4	WB11042j	China	Northern Shoveler	JX570796
5	WB11055j	China	Baikal Teal	JX570794
6	WB11058j	China	Green-winged Teal	JX570793
7	WB11098j	China	Green-winged Teal	JX570800
8	HPRS-103	UK	white broiler	Z46390
9	ADOL-7501	USA	broiler	AY027920
10	UD5	USA	broiler	AF307952
11	4817	USA	broiler	AF247385
12	ADOL-Hc1	USA	broiler	AF097731
13	10075-2	USA	broiler	GU222402
14	YZ9901	China	broiler	AY897222
15	JS-nt	China	broiler	HM235667
16	NX0101	China	broiler	AY897227
17	SD9901	China	broiler	AY897220
18	SD0001	China	broiler	AY897223
19	SD0002	China	broiler	AY897224
20	HN0001	China	broiler	AY897219
21	NM2002-1	China	broiler	HM235669
22	BJ0303	China	broiler	AY897230
23	JL08CH3-1	China	layer	HQ634809
24	HuB09JY03	China	layer	HQ634811
25	LN08SY10	China	layer	HQ634802
26	HLJ09SH01	China	layer	HQ634806
27	HLJ10SH03	China	layer	HQ634813
28	JL10HW01	China	layer	HQ634800
29	JS09GY03	China	layer	GU982308
30	NHH	China	layer	HM235668
31	SD07LK1	China	layer	FJ216405
32	sdau1002	China	layer	JN389518
33	HN1001-1	China	layer	HQ260974
34	GD1109	China	layer	JX254901
35	GD0510A	China	Chinese local chicken	EF103132
36	GD0512	China	Chinese local chicken	EF103133
37	WN100401	China	Chinese local chicken	HQ271447
38	WN100402	China	Chinese local chicken	HQ271448

a Isolates 1 to 7 are wild bird strains identified in this study. Isolates 8 to 13 are the prototype and American strains. Isolates 14 to 22 are Chinese broiler isolates. Isolates 23 to 34 are Chinese layer isolates. Isolates 35 to 38 are Chinese local chicken isolates.

## Results

### Virus isolation and identification of ALV-J

**Figure 1:**
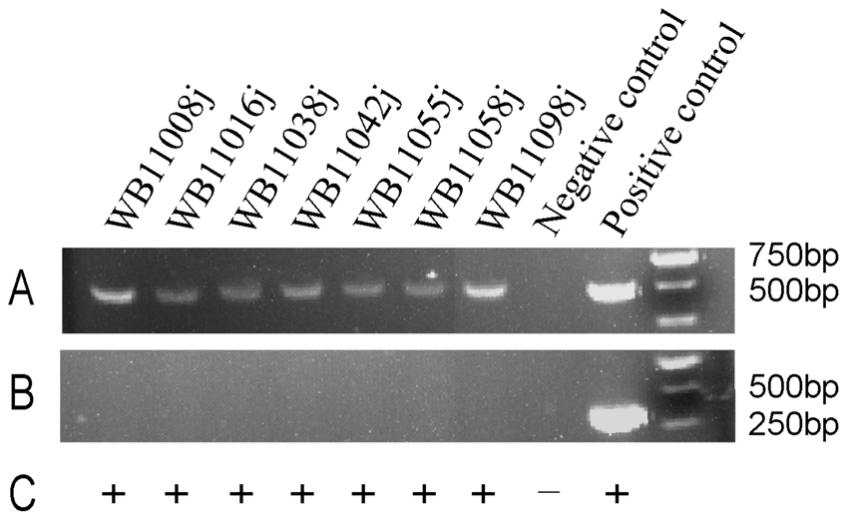
Identification of 7 ALV-J isolates by PCR (A, primer pair H5/H7; B, primer pair H5/AD1) and AC-ELISA (C). Uninfected DF-1 cells served as the negative control. DF-1 cells infected with Rous-associated virus type 1 (subgroup A) served as the positive control for PCR with primer pair H5/AD1. DF-1 cells infected with HPRS-103 served as the positive control for PCR, with primer pair H5/H7, and AC-ELISA.

A total of 7 ALV-J strains (WB11008j, WB11016j, WB11038j, WB11042j, WB11055j, WB11058j and WB11098j) were isolated from 528 clinical samples of different species of wild ducks in different provinces (Jilin, Heilongjiang, Neimeng, Liaoning, Heilongjiang, Jilin and Heilongjiang respectively)of China, which include the Eurasian Wigeon (n = 2), Northern Shoveller (n = 1), Green-winged Teal (n = 2) and Baikal Teal (n = 2). The presence of ALV in the samples was demonstrated by AC-ELISA on anti-p27 antibody-coated plates (Fig.1C). The PCRs of DNA extracted from DF-1 cells infected with any of the 7 ALV-J isolates produced an ALV-J-specific 545 bp fragment with the H5 and H7 primers (Fig.1A); however, no specific fragments were produced with the H5 and AD1 primers (Fig.1B).

### Molecular characterization of the env gene in wild duck isolates

A total of 7 ALV-J wild ducks isolates were obtained in this study; the prototype ALV-J strain HPRS-103, American ALV-J isolates, and Chinese strains from broilers, layer chickens and local chickens were used as references for comparison in the molecular studies.

The env genes of the WB11008j, WB11016j, WB11038j, WB11042j, WB11055j, WB11058j and WB11098j isolates are each 1515 nucleotides long. The nucleotide changes that occurred throughout the env gene showed a maximum divergence of 0.5% with nucleotide sequence identities ranging from 99.5% to 100%. The analysis of the deduced amino acid sequences showed that the maximum divergence in the amino acid sequence was 0.8% with sequence identities ranging from 99.2% to 100%. The wild ducks isolates were 93.2% to 93.5% identical to prototype strain HPRS-103, 93.9% to 96.1% identical to the American strains, 93.1% to 95.2% identical to Chinese broiler isolates, 93.6% to 97.9% identical to layer chicken isolates, and 93.9% to 94.5% identical to Chinese local chicken isolates at the nucleotide level.

**Figure 2:**
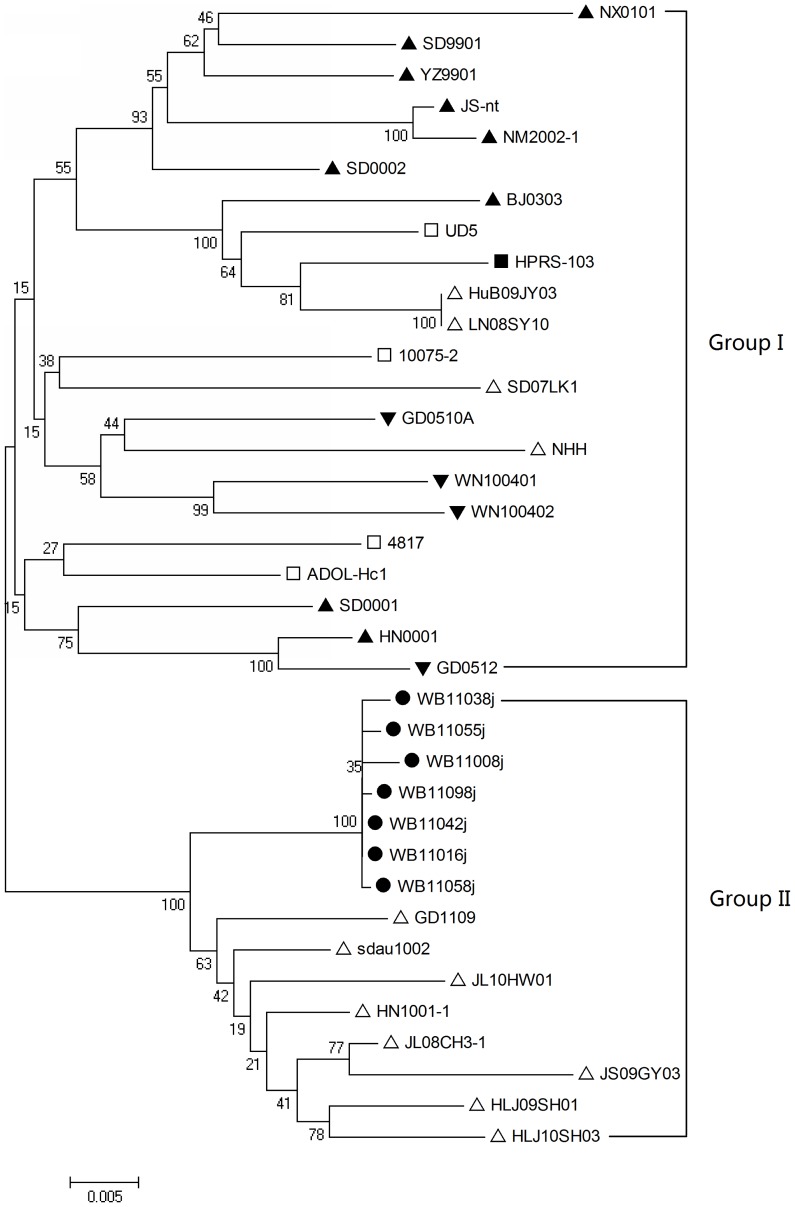
Phylogenetic analysis of the *env* gene nucleotide sequences from the ALV-J isolates and reference strains. The tree was constructed on the basis of the minimum-evolution method using MEGA 4.0 software. Bootstrap values were calculated with 1,000 replicates of the alignment. The two groups are marked. The circles represent the wild bird ALV-J isolates. The solid triangles represent the broiler ALV-J isolates. The hollow triangles represent the layer ALV-J isolates. The solid inverted triangles represent the Chinese local chicken ALV-J isolates. Triangles represent the layer ALV-J isolates. The solid square represents HPRS-103, the prototype strain of ALV-J. The hollow squares represent the American ALV-J strains from meat-type chickens.

The phylogenetic analysis indicated that of the wild ducks ALV-J isolates, WB11008j, WB11016j, WB11038j, WB11042j, WB11055j, WB11058j and WB11098j showed close homology (99.5% to 100.0%) with each other and belonged to one branch. Although there were some differences in sequence, the 7ALV-J wild ducks isolates were relatively close homologous with some layer chicken ALV-J isolates (e.g., sdau1002, GD1109 and HN1001) and were designated as group II (Fig.2). The prototype strain HPRS-103 and the American strains were relatively homologous with Chinese broiler isolates, Chinese local chicken isolates, and some layer chicken isolates (HuB09JY03, LN08SY10, NHH and SD07LK1), and were thus designated group I (Fig.2).

Compared with the reference ALV-J isolates used in this study, amino acid substitutions in all of the wild ducks isolates were distributed throughout the envelope glycoprotein sequences and no special amino acid mutation was found in gp85 and gp37. Compared with the prototype strain HPRS-103, 9 amino acid mutations (117G, 118G, 119T, 123I, 128F, 136A, 143E, 147G and 150H) were found in the hr1 region, 5 amino acid mutations (191N, 192F, 197G, 200G and 202K) were found in the hr2 region, 4 amino acid mutations (238E, 239N, 240K and 241T) were found in the vr2 region, and no amino acid mutation was found in the vr1 region.

### Molecular characterization of the 3′UTR in wild duck isolates

**Figure 3:**
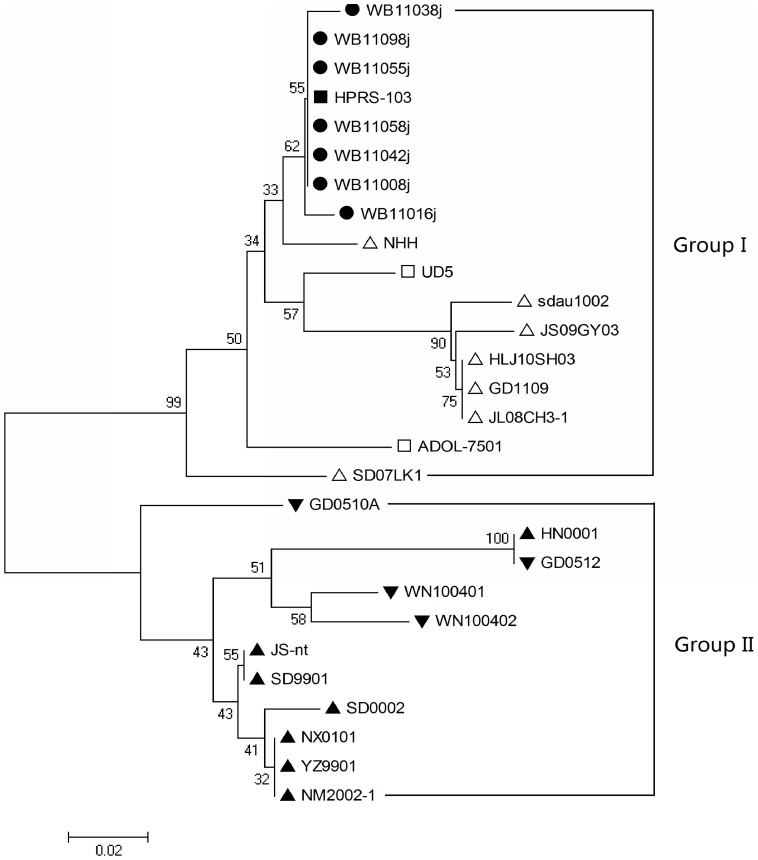
Phylogenetic analysis of the 3′UTR nucleotide sequences from the ALV-J isolates and reference strains. The circles represent the wild bird ALV-J isolates. The solid triangles represent the broiler ALV-J isolates. The hollow triangles represent the layer ALV-J isolates. The solid inverted triangles represent the Chinese local chicken ALV-J isolates. Triangles represent the layer ALV-J isolates. The solid square represents HPRS-103, the prototype strain of ALV-J. The hollow squares represent the American ALV-J strains from meat-type chickens.

A phylogenetic analysis of the ALV-J 3′UTR nucleotide sequences showed that wild ducks isolates WB11008j, WB11016j, WB11038j, WB11042j, WB11055j, WB11058j and WB11098j were very homologous (greater than 99%) with the prototype strain HPRS-103 and belonged to one branch (Fig.3). Although there were some differences in sequence, the 7ALV-J wild ducks isolates and the prototype strain HPRS-103 were relatively close homologous with layer chicken ALV-J isolates and the American strains, and they were designated as group I (Fig.3). Chinese broiler isolates (e.g., JS-nt, NX0101 and HN0001) were homologous with Chinese local chicken isolates (e.g., GD0512 and WN100401) and were designated as group II (Fig.3).

The redundant transmembrane region (rTM), the direct repeat 1 (DR-1) and the E element are located in the 3′UTR. A comparison of the nucleotide sequences of the ALV-J wild ducks isolates 3′UTRs revealed that no nucleotides were deleted in the rTM and DR-1 regions, as in the prototype strain HPRS-103.

**Figure 4:**
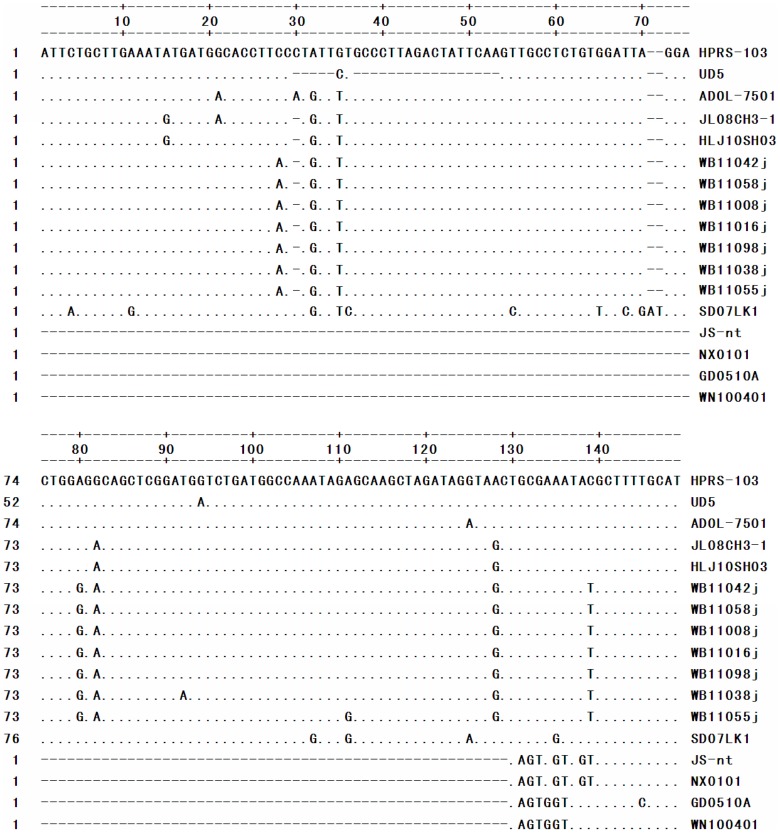
Comparison of the nucleotide sequences of the E elements. The dots indicate identical residues, while the letters indicate base substitutions. The dashes indicate gaps produced in the alignment.

The nucleotide sequences of the E elements in the ALV-J wild ducks isolates were aligned with those of the ALV-J reference strains. The sequence alignment revealed that most of the sequence of the E element was conserved in all of the wild ducks ALV-J isolates, and only minor mutations were observed (Fig.4). There was a single nucleotide deletion (bases 29 and 31). This deletion was similar to the mutation in the E element that was found in some of the layer chicken ALV-J isolates (e.g., JL08CH3-1) [8].

## Discussion

Currently, reports on the prevalence of ALV-J are limited to domestic fowl [7], [8], [16]. Our study demonstrated the presence of ALV-J in wild ducks. Seven ALV-J strains were isolated only from Eurasian Wigeon, Northern Shoveller, Green-winged Teal and Baikal Teal. However, the conclusion cannot be drawn from the above data that this four wild ducks are more susceptible to the virus than other species wild ducks. This data on ALV-J in wild ducks is not comprehensive, because the species and quantity of wild ducks used in this study was completely dependent on what samples were available at specific bird banding stations and National Terrestrial Wildlife Pathogen-origins and Epidemic Diseases Monitoring Stations. More different species, a greater quantity of wild birds and a longer continued surveillance time are essential to the understanding of the ecology of ALV-J in wild ducks. In addition, it cannot determine that wild ducks are susceptible to ALV-J infection, or they are only a natural reservoir of ALV-J. The virus might really infect wild ducks or might be ‘passing through’ wild ducks (without replication) following contact with domestic poultry carrying a very high load of virus. All of wild ducks collected in this study were dead, so we could not conduct a serological assessment of the birds for demonstrating true infection and replication.

The env glycoprotein of ALV, as in other retroviruses, functions mainly as a ligand for receptor binding so that the virus can enter susceptible cells [17]. It was also demonstrated to be a major determinant of lineage-specific oncogenicity and host range [18], [19]. Studies on other ALV subgroups have shown that the central region of the gp85 subunit contains regions of sequence variability in five clusters, designated hr1, hr2, vr1, vr2, and vr3 [20]. Analyses of ALV env genes have identified the hr1 and hr2 domains to be the principal receptor interaction determinants [21]; vr3 also plays a role in determining the specificity of receptor recognition [18]. In the present study, the env genes of wild ducks isolates were significantly different from the env genes in prototype strain HPRS-103, American strains, broiler ALV-J isolates and Chinese local chicken isolates, and they were relatively homologous with some layer chicken ALV-J isolates. An alignment of the deduced amino acid sequences of the isolates from 7 wild ducks and the reference strains revealed that some amino acid substitutions were distributed throughout the hr1, hr2 and vr2 regions when compared with the prototype strain HPRS-103. The amino acid substitutions were similar to the mutations found in many layer chicken isolates. These results suggest that these amino acid substitutions might be associated with changes in the host range and pathogenicity of ALV-J.

The 3′UTR of ALV, which contains potent regulatory sequences that influence chromosomal and viral gene expression, is important for viral pathogenesis and replication [22], [23]. Recent reports have demonstrated that the ALV 3′UTR might be the region of ALV with the greatest genetic variation because of the partial or complete deletion in the rTM, the partial deletion of the DR-1 and the deletions in the E elements that are found in some isolates [24], [25]. The 3′UTR of 7 of the wild ducks ALV-J isolates were very homologous with the prototype strain HPRS-103, and no obvious deletion was found in the 3′UTR except for a 1 bp deletion in the E element. This was rare for the ALV-J strains that have been isolated in recent years. The deletion of the rTM has been observed in a large number of ALV-J strains in recent years [25]–[27] and seemed to be a trend in the sequence variation in the 3′UTRs in current ALV-Js in China [8], [28]. The rTM is suspected to be related to the evolution and virulence of this virus, though it might not be necessary for viral replication. A previous study indicated that DR-1 elements are exclusively found in sarcoma viruses and ALV-J [29]. DR-1 may contribute to the fitness of the viruses, because these elements have been associated with the efficient accumulation of unspliced RNA in the cytoplasm and the selective increase of spliced src mRNA in avian sarcoma viruses [30]. To our knowledge, DR 1 is present in the 3′UTRs of all ALV-J isolates without any exception.

The E element had a deletion of 127 bp in earlier Chinese strains [31]. An analysis of the E element sequence revealed a 1 bp deletion in this region in wild duck isolates. The deletion introduced a specific binding site for c-Ets-1, according to a motif analysis. Although the function of this element remains unclear, it contains binding sites for the transcription factor c/EBP and may also act as an enhancer [32]. The existence of the E element in the 3′UTR increases the rate of tumor occurrence in ALV-J-infected chickens, although this element is not a decisive factor in the induction of tumors [31]. Whether these properties of the E element from wild ducks ALV-J isolates are related to their oncogenicity is unknown and should be further investigated.

ALV-Js have evolved rapidly, which results in a change in host range, first infecting meat-type chickens and then layer flocks and many local chickens in China. Our study showed that ALV-J had been detected in wild ducks. Do ALV-J strains found in wild ducks come from other wild birds or domestic fowl? How much damage will ALV-Js do to wild bird populations? Will ALV-J in wild ducks severely impact the transmission of ALV-J in domestic fowl? The answers to these questions are unknown and need to be further investigated. A longer period of continued surveillance and further study of the molecular epidemiology of ALV-J in wild birds will be essential to the understanding of the evolution and ecology of ALV-J.
